# Cluster analysis of differences in medical economic burden among residents of different economic levels in Guangdong Province, China

**DOI:** 10.1186/s12913-020-05817-y

**Published:** 2020-10-28

**Authors:** Jialong Chen, Liuna Yang, Zhenzhu Qian, Mingwei Sun, Honglin Yu, Xiaolei Ma, Chonghua Wan, Yunbin Yang

**Affiliations:** 1 School of Public Health, Guangdong Medical University, Dongguan, 523808 China; 2 Guangdong Medical University, No.1 Xincheng Blvd, Songshan Lake National Hightech Industrial Development Zone, Dongguan, 523808 Guangdong China; 3 School of Humanities and Management, Research Center for Quality of Life and Applied Psychology, Guangdong Medical University, Dongguan, 523808 China

**Keywords:** Medical economic burden, Different economic levels, Aging, Equity

## Abstract

**Background:**

This study compares and analyzes the differences of residents’ medical economic burden in different economic levels, explores the factors for improving the equity of health services in Guangdong, China.

**Methods:**

Cluster analysis was carried out in 20 cities of Guangdong Province by taking 7 key factors on the equity of health services as indicators. Seven key factors were collected from Guangdong Statistical Yearbook 2017 and the Sixth National Population Census. R-type clustering was used to reduce the dimensionality of 7 candidate variables through similarity index. Q-type clustering was used to classify 20 cities in Guangdong Province.

**Results:**

The cluster analysis divided Guangdong Province into three regions with different medical economic burden. The greater the proportion of the elderly over 65 years old, the greater the proportion of health care expenditure to per capita consumer expenditure of residents, and the heavier the medical economic burden. On average, 10.75% of the general budget expenditure of each city in Guangdong Province is spent on health care.

**Conclusions:**

The lower per capita GDP, the higher proportion of the elderly over 65 years old and the lack of medical technicians are risk factors for the heavier medical burden of the residents and the fairness of health services. While increasing the health expenditure, the government needs to further complete the reform of the medical and health system, improve the efficiency of the medical system and curb the rapid rise of absolute health expenditures of individuals, which can reduce the economic burden of residents’ medical care.

## Background

Globally, the problem of regional disparities in medical economic burden is universal [1, 2]. A review of global health financing shown that per capita health expenditure in high-income countries reached $5252 in 2016, compared with $40 in low-income countries. The former is 130 times higher than the latter, and it is estimated that the ratio will remain at a similar level by 2050, which indicates that there is obvious inequity in health services among regions with different economic levels [3]. A study in the European Union shown that the cost of health care for cancer varies greatly from 16 euros per capita in Bulgaria to 184 euros per capita in Luxembourg [4]. Similarly, China has a vast territory, and the level of economic development and medical burden vary greatly in different regions.

According to the Research Report on the Ten Years of China’s Medical and Health Reform (2009 ~ 2019), a series of important reforms have been summed up. After 20 years of development, China has established a comprehensive basic medical system. By the end of 2018, there were 1.345 billion insured people, accounting for more than 95% of the total population of China. Among them, the basic medical insurance for urban enterprise employees was gradually established in 1997, adopting the mode of “ combining socially pooled funds with personal contributions “. The new rural cooperative medical system for rural residents was pilot started in 2003, and was fully launched in 2007. Its financing mode is mainly financial subsidies, and the number of subsidies is increasing, and the overall planning area is set up with counties as the unit, making a breakthrough. For Urban Non employment residents, including the elderly, children and other non-employment groups, the pilot project started in 2007, and the basic medical insurance system for urban residents was formally established in 2011, and its financing mode and operation mechanism are similar to those of the new rural cooperative medical system. In the new rural cooperative medical system and urban residents’ insurance, public medical treatment is still retained. By 2013, China has basically realized the comprehensive coverage of the population. After 2013, China’s basic medical insurance system covering the whole people has been basically finalized, and gradually explored the city level overall planning at the overall planning level.

The report of the seventh meeting of the Standing Committee of the 13th National People’s Congress pointed out that from 2013 to 2017, the total expenditure of the national finance and health care reached 5950.2 billion yuan, with an average annual growth rate of 11.7%. Among them, the national financial and health expenditure in 2017 was 1445.1 billion yuan, an increase of 515.6 billion yuan or 55.5% compared with 2013, accounting for 7.1% of the national financial expenditure, an increase of 0.5% compared with 2013. In 2018, the national financial budget arranged medical and health expenditure of 1529.1 billion yuan, an increase of 84 billion yuan over the previous year, accounting for 7.3% of the national financial expenditure. Meanwhile, China’s per capita GDP rose from 5710.6 yuan in 2013 to 7755 yuan in 2018. However, there is a huge disparity in the medical economic burden of various regions in China.

Generally, the medical economic burden of the southeastern provinces of China is much smaller than that of other regions. Regional disparities have become the biggest challenge in further reducing the medical economic burden of residents [5]. Guangdong Province is located in the southern part of China, connecting the mainland and Hong Kong. It covers an area of 179,700 km^2^ and 21 prefecture-level cities, whose total population reached 109.99 million in 2016. Since the implementation of the reform and opening-up policy in 1978, Guangdong Province has witnessed sustained and rapid economic development and the gross domestic product (GDP) ranks first in the country for 30 consecutive years in China. However, there are also problems such as inconsistent aging process, uneven development degree, and large differences in government health investment among cities, and fail to achieve fairness in health services.

Although Chinese economy has developed rapidly in decades, so many people are suffering the great cost of medical treatment. To provide affordable, cost-effective, and equitable healthcare for all people, China has experienced a series of health reforms during the past two decades, including increase financial investment, expand medical insurance coverage, and explore reform of medical insurance payment methods [6]. The specific reform measures include paying by disease, paying by DRGs (diagnosis related groups) and strengthen basic medical and health institutions, etc. [7]

According to the statistics bulletin of China’s health development, the total health expenditure in 2010 was 1998.04 billion yuan, including 573.25 billion yuan (28.7%) of government health expenditure and 705.13 billion yuan (35.3%) of personal health expenditure. The per capita health expenditure is 1490.1 yuan. The total cost of health is 4.98% of GDP. In 2019, the total national health expenditure is expected to reach 6519.59 billion yuan, including 1742.85 billion yuan (26.7%) of government health expenditure and 1848.95 billion yuan (28.4%) of personal health expenditure. The per capita total health expenditure is 4656.7 yuan, and the percentage of total health expenditure to GDP is 6.6%. The data shows that the Chinese government’s investment in health services has increased significantly, which has effectively reduced the OOP of residents, and the residents’ consumption capacity and demand for medical services have also been effectively improved. The proportion of out-of-pocket (OOP) expenditures in total health expenditures decreased from 59.97% in 2001 to 29.27% in 2015. The health care reform policy has got certain achievements and improved residents’ sense of access [8–10].

Whether in developed or developing countries, medical economic burden is a topic worthy of attention. The incidence of global catastrophic expenditure was 9.7% in 2000, 11.4% in 2005 and 11.7% in 2010. In 2010, 808 million people worldwide suffered catastrophic medical expenditures [11]. Influenced by changes in economic growth, rapid growth in health care prices and population aging, it is expected that U.S. national health expenditure will grow at an average annual growth rate of 5.5% in 2018–2027, accounting for 19.4% of GDP in 2027 [12]. In developing countries, owing to insufficient government health expenditure, the burden of residents’ OOP expenditure is more significant [13, 14]. The proportion of health care expenditure in per capita consumption expenditure can well reflect the burden of cash expenditure of local residents in the face of disease. Per capita GDP is a classic indicator to reflect the degree of local economic development. Over 65 years old is recognized as the mark age of aging. These indicators are found in the data published by the government, which are authoritative and accurate. There are many alternative indicators, but these indicators we collected are classic and generally accepted.

Previous studies on medical economic burden mainly focused on single disease or single region, but lacked comparative studies for different levels of regional economy development [15–17]. From the perspective of health service equity, this study takes Guangdong Province as an example, compares the differences of residents’ medical economic burden in cities with different economic levels and explores the influence factors, which can provide a basis for planning and decision-making. The aim of the medical and health system reform is to provide affordable medical and health services to the residents, so that they will not suffer from inappropriate economic difficulties, which can be measured by the proportion of residents’ cash expenditure relative to their income or consumption [11]. This study uses the index of the proportion of health care expenditure to per capita consumer expenditure to measure the medical economic burden of residents, which is representative.

## Methods

### Data sources

This study collected the data of 7 variables of each city in Guangdong Province. Per capita GDP, the proportion of urban population and the number of medical technical personnel per 1000 permanent population are from Guangdong Statistical Yearbook 2017; Per capita disposable income of permanent households, proportion of expenditure for medical and health care to local government general budgetary expenditure, and the proportion of health care expenditure to per capita consumer expenditure are from the Statistical Yearbook 2017 of each city. Proportion of population over 65 years old came from the data of Guangdong Province in the Sixth National Population Census. These seven indicators cover the medical economic burden, the age composition of the population, the social composition of the population, the level of economic development, the government’s financial investment in health services and the status of human resources for health. In 2016, the average exchange rate of US dollar to RMB was 6.6423.

There are 21 cities in Guangdong province. Due to the lack of a key variable in the “Yunfu city Statistical Yearbook 2017”, this study only included 20 cities outside Yunfu city.

### Research methods

Descriptive research methods are used for statistical analysis of the data and shown as Mean ± SEM. R type clustering (variable clustering) uses Pearson correlation coefficient as similarity index. The Pearson correlation coefficient in the similarity matrix is close to 1 or - 1, which indicates that the two variables are highly correlated and can be substituted for each other. In this study, Pearson correlation coefficient is greater than 0.8 can be considered that there is a strong correlation. Q type clustering (city clustering) uses the square of Euclidean distance as the similarity index. One-way ANOVA is used to determine whether cluster variables contribute to the process and results of city clustering. Excel 2013 was used for data collection and SPSS 25.0 was used for cluster analysis of 7 indicators and 20 cities. The average value of each indicators combines with professional knowledge and analysis purposes to define and explain the various types of cities.

## Results

### General information of 7 indicators

A total of 20 cities were included in this study. Brief statistical descriptions on 7 key indicators were shown in the Table 1.

**Table 1:** General information of 7 indicators

Variables	Max	Min	Mean ± SEM
Proportion of health care expenditure to per capita consumer expenditure(%)	7.77(Maoming city)	3.46(Chaozhou city)	5.42 ± 1.36
Per capita GDP(yuan)	167,411.15(Shenzhen city)	24,031.6(Meizhou)	65,641.65 ± 43,277.34
Proportion of population over 65 years old (%)	10.35(Meizhou city)	1.79(Shenzhen city)	7.35 ± 2.56
Proportion of urban population to permanent population (%)	100(Shenzhen city)	40.8(Maoming)	64.86 ± 19.67
Proportion of expenditure for medical and health care to local government general budgetary expenditure(%)	16.42(Jieyang city)	2.03(Dongguang city)	10.74 ± 3.89
Number of medical technical personnel per 1000 permanent population	9.82(Guangzhou city)	3.5(Shanwei city)	5.77 ± 1.65
Per capita disposable income of permanent households (yuan)	48,695(Shenzhen city)	17,654.1(Jieyang city)	26,777.72 ± 11,493.39

### Results of cluster analysis of seven alternative indicators

Cluster analysis was used to reduce the dimensions of seven indicators. The results showed that the Pearson correlation coefficient between the proportion of urban population to permanent population and per capita GDP was 0.874, and that between proportion of urban population to permanent population and proportion of population over 65 years was 0.870. The Pearson correlation coefficient between per capita disposable income and per capita GDP is 0.956, and that between per capita disposable income and proportion of population over 65 years is 0.838. Pearson correlation coefficient between per capita disposable income and the proportion of urban population to permanent population is 0.936.

**Fig. 1:**
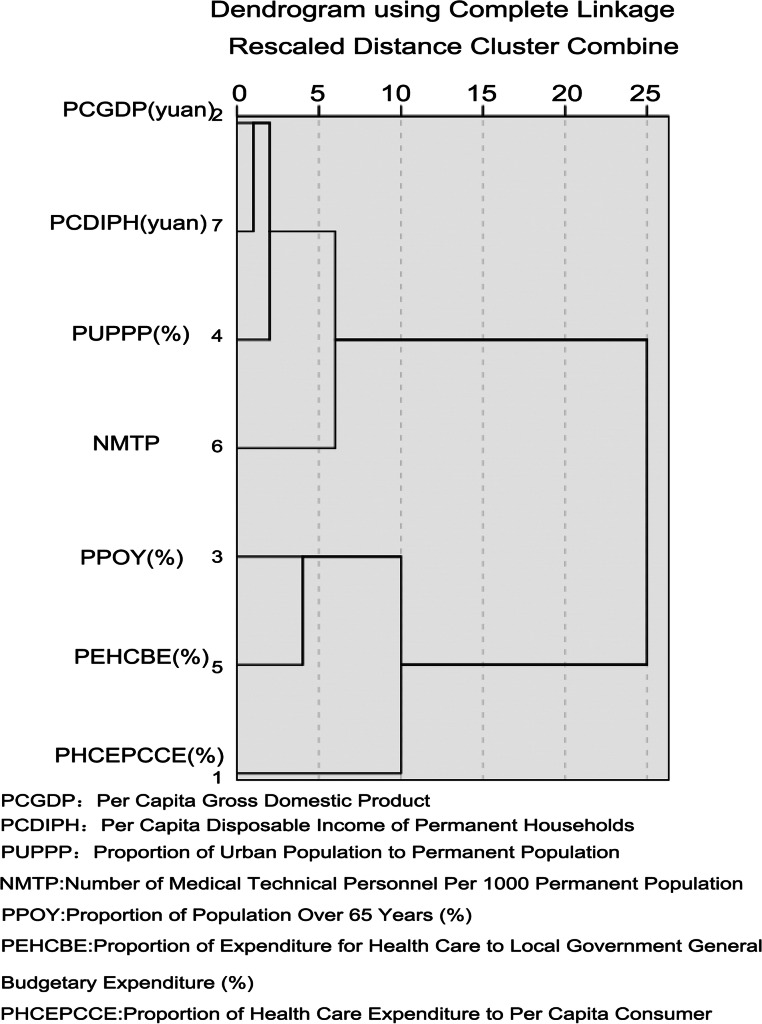
Dendrogram of Cluster Analysis of Indicators

Therefore, we excluded indicators as follows: the proportion of urban population to permanent population, per capita disposable income. The selected indicators include per capita GDP, the proportion of population over 65 years, the proportion of health care expenditure to per capita consumer expenditure, the proportion of expenditure for medical and health care to local government general budgetary expenditure, and the number of medical technical personnel per 1000 permanent population (Table 2 and Fig. 1). The selected indicators were used in cluster analysis of 20 cities.

**Table 2:** Similarity Matrix for Cluster Analysis of Indicators

	Proportion of Health Care Expenditure to Per Capita Consumer Expenditure (%)	Per Capita Gross Domestic Product (yuan)	Proportion of Population Over 65 Years (%)	Proportion of Urban Population to Permanent Population (%)	Proportion of Expenditure for Medical and Health Care to Local Government General Budgetary Expenditure (%)	Number of Medical Technical Personnel Per 1000 Permanent Population	Per Capita Disposable Income of Permanent Households (yuan)
Proportion of Health Care Expenditure to Per Capita Consumer Expenditure (%)	1.000	−.456	.487	−.578	.276	−.153	−.447
Per Capita Gross Domestic Product (yuan)	−.456	1.000	−.756	**.874**	−.668	.777	**.956**
Proportion of Population Over 65 Years (%)	.487	−.756	1.000	**−.870**	.674	−.315	**−.838**
Proportion of Urban Population to Permanent Population (%)	−.578	**.874**	**−.870**	1.000	−.739	.563	**.936**
Proportion of Expenditure for Medical and Health Care to Local Government General Budgetary Expenditure (%)	.276	−.668	.674	−.739	1.000	−.515	−.760
Number of Medical Technical Personnel Per 1000 Permanent Population	−.153	.777	−.315	.563	−.515	1.000	.723
Per Capita Disposable Income of Permanent Households (yuan)	−.447	**.956**	**−.838**	**.936**	−.760	.723	1.000

### Results of cluster analysis of twenty cities

**Fig. 2:**
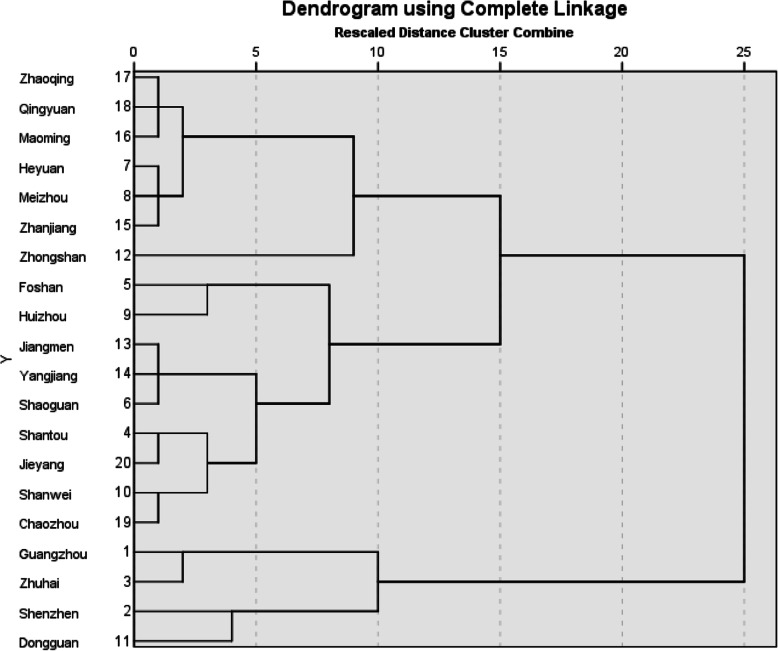
Dendrogram of Cluster Analysis of Cities

The 20 cities can be divided into three categories by cluster analysis. The first category is the areas with lighter medical economic burden and better fairer medical and health services, including Guangzhou, Shenzhen, Zhuhai and Dongguan; the second category is the areas with medium medical economic burden and fairer medical and health services, including Shantou, Foshan, Shaoguan, Huizhou, Shanwei, Jiangmen, Yangjiang, Chaozhou and Jieyang. The third category is the areas with heavy medical economic burden and poor fairer medical and health services, including Heyuan, Meizhou, Zhongshan, Zhanjiang, Maoming, Zhaoqing and Qingyuan (Table 3 and Fig. 2).

**Table 3:** Classification results of cities by cluster analysis

City	Clusters
1:Guangzhou	1
2:Shenzhen	1
3:Zhuhai	1
4:Shantou	2
5:Foshan	2
6:Shaoguan	2
7:Heyuan	3
8:Meizhou	3
9:Huizhou	2
10:Shanwei	2
11:Dongguan	1
12:Zhongshan	3
13:Jiangmen	2
14:Yangjiang	2
15:Zhanjiang	3
16:Maoming	3
17:Zhaoqing	3
18:Qingyuan	3
19:Chaozhou	2
20:Jieyang	2

### One-way analysis of variance (ANOVA) of five selected indicators

In order to determine whether the indicators used for clustering contribute to the results of the clustering process, one-way ANOVA was used in this study. The results shown that the *P* values of the five indicators are significant different. Therefore, the five indicators were used for cluster analysis of 20 cities, and it is reasonable to use them as the indicators for cluster analysis (Table 4).

**Table 4:** One-way ANOVA of five selected indicators

		Sum of Squares	df	Mean Square	F	Sig.
Proportion of Health Care Expenditure to Per Capita Consumer Expenditure (%)	Between Groups	27.565	2	13.783	29.910	.000
	Within Groups	7.834	17	.461		
	Total	35.399	19			
Per Capita Gross Domestic Product (yuan)	Between Groups	21,941,875,551.721	2	10,970,937,775.861	13.670	.000
	Within Groups	13,643,762,457.801	17	802,574,262.224		
	Total	35,585,638,009.522	19			
Proportion of Population Over 65 Years (%)	Between Groups	61.955	2	30.977	8.291	.003
	Within Groups	63.514	17	3.736		
	Total	125.469	19			
Proportion of Expenditure for Medical and Health Care to Local Government General Budgetary Expenditure (%)	Between Groups	141.400	2	70.700	8.173	.003
	Within Groups	147.061	17	8.651		
	Total	288.460	19			
Number of Medical Technical Personnel Per 1000 Permanent Population	Between Groups	23.311	2	11.656	6.907	.006
	Within Groups	28.686	17	1.687		
	Total	51.997	19			

### Comparison of the means of three clusters of cities

The average proportion of health care expenditure to per capita consumer expenditure in the first, second and third categories are 4.24, 4.73 and 7.01%, respectively. The higher the per capita GDP and the lower the proportion of health care expenditure to per capita consumer expenditure, the lighter the medical economic burden and the better the equity. The greater the proportion of the elderly over 65 years old, the greater the proportion of health care expenditure to per capita consumer expenditure of residents, and the heavier the medical economic burden. On average, 10.75% of the general budget expenditure of each city in Guangdong Province is spent on health care. Generally speaking, the increase of government health expenditure in the proportion of fiscal expenditure has not contributed to the reduction of residents’ medical economic burden. In areas where health technicians are scarce, the medical economic burden is also heavy (Table 5).

**Table 5:** The means of five indicators in three clusters of cities

Clusters		Proportion of Health Care Expenditure to Per Capita Consumer Expenditure (%)	Per Capita Gross Domestic Product (yuan)	Proportion of Population Over 65 Years (%)	Proportion of Expenditure for Medical and Health Care to Local Government General Budgetary Expenditure (%)	Number of Medical Technical Personnel Per 1000 Permanent Population
1	Mean	4.2350	131,643.6628	3.9400	5.4375	7.9125
	Std. Deviation	.67060	35,547.38186	2.30527	3.12486	2.00086
2	Mean	4.7300	51,934.8097	7.7911	12.2178	5.0911
	Std. Deviation	.76274	27,364.83806	1.74819	2.70893	1.35889
3	Mean	7.0086	45,549.3076	8.7486	11.8914	5.4314
	Std. Deviation	.55231	25,371.36998	1.96308	3.13741	.56322
Total	Mean	5.4285	65,641.6546	7.3560	10.7475	5.7745
	Std. Deviation	1.36495	43,277.34183	2.56975	3.89642	1.65430

## Discussions

### The higher the per capita GDP, the lighter the medical economic burden

**Fig. 3:**
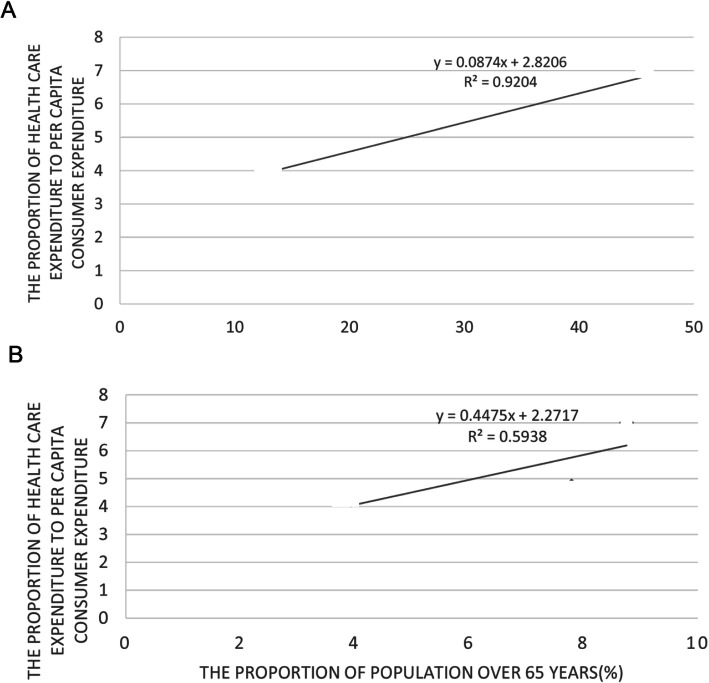
Linear regression models

In order to describe the relationship between the per capita GDP and the lighter the medical economic burden, we have constructed linear regression model (Fig. 3a). This result is consistent with the conclusions of Li Liqing et al. The proportion of urban residents’ health care expenditure to their per capita consumer expenditure is smaller than that of rural residents [18]. On the one hand, there is a general rule that residents’ health care expenditure increases with the increase of income level. In areas with higher economic income, residents have stronger health awareness and higher per capita health expenditure, but at the same time, this part of residents’ per capita consumer expenditure is also high, so the denominator is larger [19]. On the other hand, the number of grass-roots health institutions in areas with low economic development level is small and the accessibility of preventive health care services is poor, therefore, many patients are not treated in time in the early stage, which leads to the increase of medical expenses in the later stage, resulting in a higher medical economic burden. This means that under current health policy, low-income groups benefit less than high-income groups [20].

Since the year 2000, a series of reforms have been made in Switzerland, which including the MHI system, the financing of hospitals, the area of pharmaceuticals, the control of epidemics and harmonized regulation of human resources. However, it is difficult to making health reforms in Switzerland as a very broad consensus of the main stakeholders is required. Yet, it is very successful at negotiating compromises that are supported (or at least not opposed) by all relevant stakeholders. This leads to lengthy reform processes [21].

In France, the overall health of the population and the level of economic protection provided by the health system are very good. Public financing of health care spending is the highest in Europe, while OOP spending is one of the lowest. Recent reforms have focused on addressing barriers to access to health care and improving chronic and long-term care to better meet the needs of the population. Recent reforms have focused on addressing barriers to access to health care and improving chronic and long-term care to better meet the needs of the population [22]. (France: Health System Review)

### The greater the proportion of the age of over 65 years, the heavier the medical economic burden

In order to describe the relationship between the proportion of the age of over 65 years and the medical economic burden, we have constructed linear regression model (Fig. 3b). Several studies have shown that the increase in the proportion of people over 65 years of age may lead to an increase incidences of chronic diseases and the demand for health care, thus affecting health expenditure [23–25]. In addition to being susceptible to chronic diseases, the elderly are also more likely to suffer from comorbidities [26], which causes them to consume more medical expenses than other age groups [27]. Therefore, in areas with a large proportion of the elderly over 65 years old, the medical economic burden of residents is also heavier.

According to data released by the China Health Commission, the average life expectancy in China was 77 years in 2018, but the healthy life expectancy was only 68.7 years, that is, residents had 8.3 years to live with illness. This shows that the elderly in China have a high prevalence rate, accompanied by early onset and long course of disease. If effective health interventions can be implemented to extend the healthy life expectancy of the elderly, it will effectively reduce the social medical economic burden.

### In areas where government health expenditure accounts for a high proportion of fiscal expenditure, it fails to reduce the medical economic burden

The report, Fairness and Accountability: Engaging in Health Systems in the Middle East and North Africa (MENA), highlights how MENA governments spend on average only 8% of their individual budgets on health care compared to an average of 17% spent by Organization for Economic Co-operation and Development (OECD) countries. MENA households end up paying the difference in OOP expenses reaching 40% of total health expenditure compared to 14% in OECD countries. As a result, many people end up foregoing or delaying much needed medical care because of the unaffordable and impoverishing costs.

Obviously, there is still a gap between the scale of health expenditure in Guangdong Province and the high-income areas such as OECD members. Increasing government health expenditure is important and necessary for basic health services [3]. Meanwhile, the government needs to reform the drug procurement system and medical insurance payment system to curb the dramatic increase of individual absolute health expenditure in order to truly reduce the direct burden of residents [28].

### Generally speaking, in areas where health technicians are scarce, the medical economic burden is also heavy

Adequate health human resources often mean higher medical skills and better health outcomes for residents [29, 30], which ensures timely and effective diagnosis and treatment of residents to avoid minor diseases into major diseases and cross-city medical treatment. A series of reforms were explored include: encouraging commercial insurance products, enlarging fund pool, activating medical insurance stock fund, improving quality and efficiency and promoting DRGs based payment reform. Moreover, nationwide trials of serious illness insurance for both urban and rural residents was carried out and standardizing the order of drug circulation to prevent drug price from being excessively high. Also, the level of diagnosis and treatment and medical facilities in primary-level medical and health institutions were improved through multiple strategies. Developing regional economy, paying attention to the construction of grass-roots medical institutions in economically backward areas, increasing the allocation of health human resources, and improving the quality and accessibility of medical services. To meet the basic medical needs of residents is conducive to fairness.

### Limitation

Although the indicators included in this study can reflect the level of economic development, the level of aging, the allocation of medical resources, the level of medical economic burden in Guangdong Province, we need to include more detailed and internationally recognized indicators in further research.

## Conclusion

Lower per capita GDP, higher proportion of elderly people over 65 years old and lack of medical technicians are the risk factors of heavy medical economic burden and poor equity of health services. Compared with the high-income countries, the scale of government health expenditure in Guangdong Province still lags behind. Moreover, the increase of government health expenditure has failed to alleviate residents’ medical economic burden. It is necessary to further complete the reform of the medical and health system, explore ways to improve the efficiency of the medical system, reduce the medical economic burden of residents, and improve the fairness of health services. There are still gaps for various classes or people to obtain health resources or access health services. Our work found that the equity of health services in different economic level areas of Guangdong is still insufficient. The proportion of health care expenditure to consumption expenditure is higher in the areas with poor economic development, which means that the economic burden of medical treatment is heavier.

At present, the compensation of medical insurance for urban and rural residents is limited, the demand for medical service utilization for low-income groups is insufficient, and the reduction of medical economic burden is not obvious, so the proportion of reimbursement should be increased appropriately. The aging population also has a huge impact on the medical insurance funds. We should establish a special medical security system for the elderly.

## Data Availability

All data in the study can be accessed from the corresponding author up on request.

## Abbreviations

GDP: Gross Domestic Product
OOP: Out-of-Pocket
ANOVA: Analysis of Variance
MENA: Middle East and North Africa
OECD: Organization for Economic Co-operation and Development

## References

[1] Bauchner H, Fontanarosa PB (2018). Health care spending in the United States compared with 10 other high-income countries: what Uwe Reinhardt might have said. JAMA.

[2] Parente ST (2018). Factors contributing to higher health care spending in the United States compared with other high-income countries. JAMA.

[3] Global Burden of Disease Health Financing Collaborator Network (2019). Past, present, and future of global health financing: A review of development assistance, government, out-of-pocket, and other private spending on health for 195 countries, 1995-2050. Lancet.

[4] Luengo-Fernandez R, Leal J, Gray A, Sullivan R (2013). Economic burden of cancer across the European Union: a population-based cost analysis. Lancet Oncol.

[5] Dou G, Wang Q, Ying X (2018). Reducing the medical economic burden of health insurance in China: achievements and challenges. Biosci Trends.

[6] Yip WC, Hsiao WC, Chen W, Hu S, Ma J, Maynard A (2012). Early appraisal of China's huge and complex health-care reforms. Lancet.

[7] Zeng JQ (2019). The pilot results of 47 148 cases of BJ-DRGs-based payment in China. Int J Health Plann Manag.

[8] Meng Z, Zhu M, Cai Y, Cao X, Wu H (2019). Effect of a typical systemic hospital reform on inpatient expenditure for rural population: the Sanming model in China. BMC Health Serv Res.

[9] Li L, Fu H (2017). China’s health care system reform: Progress and prospects. Int J Health Plann Manag.

[10] Deng J, Tian H, Guo Y, Ma T, Sun Y, Zhang S, Yang T, Tian X (2018). A retrospective and prospective assessment of the zero-markup drug reform in China from the perspective of policy diffusion. Int J Health Plann Manag.

[11] Wagstaff A, Flores G, Hsu J, Smitz MF, Chepynoga K, Buisman LR, van Wilgenburg K, Eozenou P (2018). Progress on catastrophic health spending in 133 countries: a retrospective observational study. Lancet Glob Health.

[12] Sisko AM, Keehan SP, Poisal JA, Cuckler GA, Smith SD, Madison AJ, Rennie KE, Hardesty JC (2019). National health expenditure projections, 2018-27: economic and demographic Trends drive spending and enrollment growth. Health Aff (Millwood).

[13] Chowdhury S, Gupta I, Trivedi M, Prinja S (2018). Inequity &amp; burden of out-of-pocket health spending: district level evidences from India. Indian J Med Res.

[14] Global Burden of Disease Health Financing Collaborator Network (2017). Evolution and patterns of global health financing 1995-2014: development assistance for health, and government, prepaid private, and out-of-pocket health spending in 184 countries. Lancet.

[15] Xiong M, Li M, Zheng D, Wang X, Su T, Chen Y, Yang B (2017). Evaluation of the economic burden of leprosy among migrant and resident patients in Guangdong Province, China. BMC Infect Dis.

[16] Putri W, Muscatello DJ, Stockwell MS, Newall AT (2018). Economic burden of seasonal influenza in the United States. Vaccine.

[17] Ding D, Lawson KD, Kolbe-Alexander TL, Finkelstein EA, Katzmarzyk PT, van Mechelen W, Pratt M (2016). The economic burden of physical inactivity: a global analysis of major non-communicable diseases. Lancet.

[18] Liqing L, Fuyi D, Lu Z, Qiaoyan L (2016). Analysis of the influence and mechanism of residents’ income change on health expenditure in China. Health Economy in China.

[19] Xiuzhe M (2017). A comparison of consumption income between urban and rural areas in Shandong Province. Contemp Econ.

[20] Pan J, Tian S, Zhou Q, Han W (2016). Benefit distribution of social health insurance: evidence from china's urban resident basic medical insurance. Health Policy Plan.

[21] De Pietro C, Camenzind P, Sturny I, Crivelli L, Edwards-Garavoglia S, Spranger A, Wittenbecher F, Quentin W (2015). Switzerland: health system review. Health Syst Transit.

[22] Chevreul K, Berg BK, Durand-Zaleski I, Hernandez-Quevedo C (2015). France: health system review. Health Syst Transit.

[23] Lopreite M, Mauro M (2017). The effects of population ageing on health care expenditure: a Bayesian VAR analysis using data from Italy. Health Policy.

[24] Harris A, Sharma A (2018). Estimating the future health and aged care expenditure in Australia with changes in morbidity. PLoS One.

[25] Duan W, Zheng A, Mu X, Li M, Liu C, Huang W, Wang X (2017). How great is the medical burden of disease on the aged? Research based on “system of health account 2011”. Health Qual Life Outcomes.

[26] Picco L, Achilla E, Abdin E, Chong SA, Vaingankar JA, McCrone P, Chua HC, Heng D, Magadi H, Ng LL (2016). Economic burden of multimorbidity among older adults: impact on healthcare and societal costs. BMC Health Serv Res.

[27] Wang C, Li F, Wang L, Zhou W, Zhu B, Zhang X, Ding L, He Z, Song P, Jin C (2018). The impact of population aging on medical expenses: a big data study based on the life table. Biosci Trends.

[28] Shi J, Liu R, Jiang H, Wang C, Xiao Y, Liu N, Wang Z, Shi L (2018). Moving towards a better path? A mixed-method examination of China's reforms to remedy medical corruption from pharmaceutical firms. BMJ Open.

[29] Anand S, Fan VY, Zhang J, Zhang L, Ke Y, Dong Z, Chen LC (2008). China’s human resources for health: quantity, quality, and distribution. Lancet.

[30] Lassi ZS, Musavi NB, Maliqi B, Mansoor N, de Francisco A, Toure K, Bhutta ZA (2016). Systematic review on human resources for health interventions to improve maternal health outcomes: evidence from low- and middle-income countries. Hum Resour Health.

